# Monthly Summary

**Published:** 1896-12

**Authors:** 


### Monthly Summary.

TREATMENT OF ANTRUM DISEASE.—Dr. John E. Bacon, describes his method of operation. It consists in cleansing and medicating the cavity through a small puncture in its inner wall in the inferior meatus of the nose, which can be made without general anesthesia and without pain. The instruments are a steel trocar and canula, two silver tubes, a silver wash tube, and a hard rubber syringe with rubber tube connections made to fit the canula and wash tubes. Cleanse the nares with the antiseptic spray, cocainize the inferior turbinal and floor on the side to be operated upon, insert a rubber operating speculum well into the nostril, and place the trocar beneath the inferior turbinal about one and one-fourth inches from the skin margin; by bending the septum to the opposite side the point of the trocar will point obliquely into the cavity of the antrum. A slight tap with a leaden or raw-hide mallet will cause the trocar to penetrate the thin bone which constitutes the inner wall of the cavity. Care must be taken not to penetrate too deeply and so wound the opposite side of the antrum, as serious hemorrhage might result. In most cases the trocar can be pushed through the thin bony wall with the fingers alone, and this should be done, when possible, to avoid the mental shock which the blow with the mallet sometimes gives. The trocar may now be withdrawn, leaving the canula in place, and the rubber tube may be attached to the canula and the cavity syringed out with warm sterilized normal salt solution. The fluid will escape into the nose through the ostium maxillare and bring with it pus if any be present. After the cleansing, the trocar may be replaced and the nut removed, when the canula may be withdrawn over the trocar; now a silver tube is slipped over the trocar and the latter is withdrawn, leaving the silver tube in place, and this may remain as long as required without any irritation. This tube is exactly fitted by the silver wash tube, and the cleansing may be repeated without inconvenience. A solution of menthol and camphor in liquid abolene may be easily sprayed through the tube and aristol or other non-irritant powder may be blown into the antrum by the same means. It is imperative to thoroughly sterilize all instruments used, and to use only warm sterilized fluid in each case, to prevent infection.—*The American Therapist*

---

A VALUABLE HINT.—"My husband," said a physician's wife not long ago, "chanced to see one day, standing on the shelf outside our kitchen window, some mould of jelly cooling for the night's dinner. They were uncovered, as they were out of the reach of cats, and in full view of the cook's watchful eye; but he questioned me about them, and asked if it was our usual custom to leave jelly thus unprotected. I was obliged to reply that, so far as I knew, it was. 'Then, he said, don't you know that when we medical men want to secure minute organisms for investigation, we expose gelatine to the air or in places where we have confined malignant germs? The gelatine speedily attracts and holds them. I'm afraid your flavored gelatine does the same. Cool the jelly if you must, but cover it with a piece of close muslin." And we have always done that since then."

It is to be feared that kitchen processes are sources of illness more often than is imagined. In many city houses the little kitchen annex where stands the refrigerator, and where various eatables are kept, is directly against a drain. Yet here stand daily uncovered milk, butter, often custards and puddings and various other absorbents. The average cook is absolutely ignorant of sanitary cause and effect, and the eternal vigilance of the house mother is the family's chief safeguard.—"Boston Journal of Commerce."

---

I DESIRE to caution young men not to be heroic and cut down and destroy a better natural crown than the artificial one put in its place.—Dr. W. N. Morrison, "Dental Review."

INTERESTING EXPERIENCE.—By A. Rose, L. D. S., Peterborough, Ont. I noticed in a recent number of this journal an experience of a peculiar nature in connection with an aluminum crown set with amalgam. The circumstances there described so nearly accorded with an experience of my own that I feel encouraged to report mine also. Having been requested by a lady patient to do something to save the root of a lower right second bicuspid from the necessity of extraction, and as quickly and cheaply as possible, I thought of making a crown of a piece of aluminum tubing about the diameter of the root, trimmed and fitted to it and filled, and cusps built on with amalgam. This I did in a short time, and sent the patient away much pleased with my efforts to replace her lost grinder. You may imagine my surprise when the lady returned next morning with a few scraps of something that looked like acid-eaten iron, about the shape of the piece of tubing used to form the crown, but ready to crumble to pieces in her hand. She said a few minutes after she had left the office she felt it getting hot and a boiling sensation about the gum, and then the filling seemed to boil and crumble away out of the crown. I concluded that some chemical action took place on the union of amalgam and aluminum in the saliva around them, but do not yet think I clearly understand the reason for the occurrence. I set to work again and with tubing, hammer and anvil soon fitted another band to the root, but this time filled it with oxy-phosphat, and find it giving good service and no apparent inclination to give way at any point. Can any one give a satisfactory explanation of the destructive agency in the first case ? If so, I, for one will be pleased to consider it, and thank him for his trouble.—*Dominion Dental Journal.*

---

NEW LOCAL ANÆSTHETIC.—An excellent local anesthetic, the effect of which lasts about five minutes, as follows :

| | |
|---|---|
| Chloroform - - - - - | 10 parts |
| Ether - - - - - - | 15 " |
| Menthol - - - - - - | 1 " |

Apply as a spray to the surface, carefully guarding the nostrils from its fumes.—*Zahnarztliches Wochenblatt.*

CONGENITAL TEETH.—Ballantyne (*Edinburgh Med. Jour.*, May, 1896) delivered a child in 1894 and a few days after birth found that two lower central incisors were cut. They resembled in their character teeth discolored by the use of iron tonics. Two new central incisors appeared in their place about the seventh month. The mother believed that the earlier pair were absorbed; Ballantyne thinks it more probable that they simply dropped out. The child remains healthy. Buist in 1893 detected the two lower central incisors already cut in a child born at term. The gum was swollen and the teeth loose. Both came out within a month and have not been replaced, although the dentition is otherwise normal. Vargas, of Barcelona, in 1895 examined an infant two days old, suffering from tongue-tie and a projection from the lower gum a little to the right of the middle line. It was cut away under cocain and proved to be an extra-alveolar dental sac containing an incisor with no root. The literature of the subject is reviewed by Ballantyne. Congenital teeth are usually lower incisors, seldom upper incisors, and very rarely molars. Cases like that reported by Vargas and published by Ballantyne undoubtedly represent ectopia of the dental follicle. The majority simply signify premature development of the teeth. Congenital teeth interfere with suckling and are ill-developed; they should therefore be removed. They have little if any relation to the health of the infant.—*British Medical Journal.*

---

INFLUENCE OF CONDIMENTS ON DIGESTION.—The action of condiments when taken with food is not definitely understood, though it is generally understood to be beneficial, as promoting digestion. An observer named Gottlieb has recently confirmed this view to some extent, as the result of experiments on rabbits. A canula being introduced into the pancreatic duct, pepper or mustard was allowed to pass into the stomach, and the secretion of pancreatic juice was found to increase to three or four times the normal quantity. It appeared more watery than usual, but possessed the same digestive powers as ordinarily.—*Phar. Journal.*

WHY AMALGAMS FAIL.—An extract from a lecture by Prof. Charles Steel, on "Metals and their Behavior when Alloyed."—Let me impress upon you to use only metals of known degrees of purity. I have frequently called your attention to the remarkable results produced by combining metals—how the union of two soft metals will sometimes produce a stiff, refractory alloy; how two metals, behaving well in the mouth separately, may, when alloyed, be acted upon most disastrously. A very small per cent, of some metals will frequently change the entire nature of an alloy. We may doubtless account for the apparently inexplicable failures of good amalgams under some circumstances by the impurity of the mercury used. All native mercury contains lead in varying proportions. It is most difficult to extract this metal entirely from the mercury. Lead, even in minute quantities, exercises a most deleterious effect on amalgam fillings, and indeed on nearly all the finer metals used by the dentist. See to it, then, that the mercury you use in your amalgam is as absolutely pure as can be obtained. While speaking of mercury, it just here occurs to me that this metal may in another way cause the variable results sometimes obtained from the same alloy. You know that when we undertake to combine the mercury with the alloy, it seems almost impossible to get a perfect solution without using an excess of mercury, which excess is frequently pressed from the mass. It is doubtful whether it is mercury alone which is thus pressed out, but more than likely that it is combined with some portions of the other metals. It has greater affinity for some metals than others, hence brings away different proportions; so if too great an amount of mercury is used, we may seriously injure a nicely adjusted alloy.—*Bi-monthly Bulletin.*

---

TOOTHACHE DROPS.—Equal parts of carbolic acid crystallized, camphor, chloral hydrate, menthol and glycerin. Pulverize separately the camphor and chloral, mix, and when liquefied add the menthol, previously triturated, and lastly the carbolic acid and glycerin liquefied together by heat. In packing the tooth cavity with this, none of the fluid should be allowed to ooze over the gums.—*Western Druggist.*

WHITE CARIES.—A word about what may cause this too prevalent evil of white decay, and the habits of people who present this condition; I find this condition usually in mouths subjected to uncleanliness, persons who are in the habit of lunching between meals, and cleansing their teeth in the morning before breakfast, or when they "dress up," but the white decay is rare where the teeth are thoroughly cleansed just before retiring. The soft foods—or foods classed as hydrocarbonates—when allowed to remain on and between the teeth, especially at night, are in my opinion responsible for much of this white decay. Such foods form the hot-bed of decay and the base of supply for lactic acid.

In the past year I have questioned patients presenting this condition, and invariably find that they do not cleanse their teeth at night just prior to retiring; that they are in the habit of lunching or are habitual customers of candy, or make a practice of taking a glass of milk on retiring. The girls at the type-writer; the maids in the kitchen; the mother with a number of children; the lean, slim person; the children who are continually munching on gingerbread with a cookie in hand, represent the class in which the greatest per cent. of this white decay is to be found. Where there is a general tendency to the conditions named, I think it improper to attempt filling of a permanent character. If the condition is allowed to remain, decay will be sure to follow in the near future and loss of fillings ensue. Do not attempt to fill teeth suffering with white decay until you are convinced that their surroundings are changed, or will be. Explain to patients the necessity of properly cleansing their teeth and the benefits to be derived therefrom. Get them to promise to spend at least three minutes a day in cleansing their teeth, and if they cannot cleanse them but once a day, let that once be just before retiring for the night, thereby having the mouth clean for the greater number of hours of the twenty-four. Under these circumstances, we may expect more of our fillings and our work in general to be a credit to us and a joy to our patients.—Dr. I. C. EDGERTON, in *Odontographic Journal.*

Peculiar Affection of the Mucous Membrane of the Lips and Mouth.—Dr. J. A. Fordyce, New York, read before the American Dermatological Association a paper entitled as above, in which he reported a case of a peculiar mottling of the lips and mucous surface of the cheeks, more noticeable when the parts were put upon the stretch. The condition had existed for two years without subjective symptoms, but the patches had gradually increased in area. The microscope showed a degeneration of the protoplasm of the epithelial cells. The muciparous glands were not involved.

Dr. Morrow said it was an interesting point that the same condition had been found in several members of the patient's family. The nature could be determined only by the microscope.

Dr. White had seen superficial changes suggestive of the case reported.

Dr. Bowen thought there might be a plugging of the glands by the process described, which would account for the yellowish or whitish bodies seen beneath the surface. He had seen bullæ followed by atrophy and attended with the formation of bodies similar to those by plugging up of glandular structures.—*Medical Record.*

---

The Hat worn by Napoleon at Eylau was sold in Paris in 1835 for $400. The coat worn by Charles XII at the battle of Pultowa brought over $100 000. A wig that once belonged to Sterne, the great English writer, was sold at public auction in London a few years ago for $3,050. In 1818 a tooth of Sir Isaac Newton was purchased by a nobleman for $3,650. The buyer had a costly diamond removed from his favorite ring and the tooth set in its place.—*St. Louis Republic.*

---

Investing Material.—Mr. Girdler uses the dust of burnt anthracite coal, instead of sand or asbestos, in conjunction with plaster, for investing cases for soldering. With this soldering composition he finds that the plaster rarely cracks.—*Ash's Quarterly.*

DEATH THREE DAYS AFTER CHLOROFORM ADMINISTRATION.—The report of a case reaches us from Germany of a patient who died from the effects of chloroform used as an anæsthetic for the removal of fourteen stumps. About seventy cubic centimeters were administered, and the patient vomited many times after the anæsthesia. On the next day the vomiting persisted and there was slight jaundice. The pulse began to be accelerated, and on the following day the urine contained albumen. On the next day the patient became restless, and the pulse rapidly increased in frequency. The pupils were widely dilated. On the third day death supervened. At the necropsy there was recent fatty degeneration of the heart, liver and both kidneys. This, of course, was a case in which chloroform should on no account have been employed; in fact we heartily condemn its use in dental practice altogether. The fatality differs from the ordinary run, inasmuch as the chloroform narcosis was not immediately fatal.—"British Journal of Dental Science."

---

TREATMENT OF PULPLESS TEETH.—Secure free access using sulfuric acid if necessary; remove contents carefully, washing out with warm water, pump beechwood creosote through till it appears at the fistulous opening; treat with thymol or cassia, applying the electric current by means of an electric wire attached to the alternating current, apply the current again and again till every particle of oil is absorbed by the tooth. Fill the apical part of the root with a paste composed of alum, thymol, glycerol and oxid zinc. Work it well up and follow with a small piece of heated gutta-percha.—Adam Flickinger, in *Cosmos*.

---

DENTAL INSPECTORS FOR SCHOOLS.—The Ontario Board of Health recently adopted the following resolution: "That dental inspectors be appointed by local boards of school trustees to periodically visit schools and examine children's teeth, and that a dental hospital be started in Toronto for the benefit of poor children; and these recommendations be urged upon the attention of the minister of education."—*Medical Mirror*.

